# Neural correlates of weight-shift training in older adults: a randomized controlled study

**DOI:** 10.1038/s41598-023-46645-4

**Published:** 2023-11-10

**Authors:** Veerle de Rond, Nicholas D’Cruz, Femke Hulzinga, Christopher McCrum, Sabine Verschueren, Jean-Jacques Orban de Xivry, Alice Nieuwboer

**Affiliations:** 1 Neuromotor Rehabilitation Research Group, Department of Rehabilitation Sciences, KU Leuven, Leuven, Belgium; 2 Motor Control and Neuroplasticity Research Group, Department of Kinesiology, KU Leuven, Leuven, Belgium; 3 Department of Nutrition and Movement Sciences, NUTRIM School of Nutrition and Translational Research in Metabolism, Maastricht University, Maastricht, The Netherlands; 4 Research Group for Musculoskeletal Rehabilitation, Department of Kinesiology, KU Leuven, Leuven, Belgium; 5 Leuven Brain Institute (LBI), Leuven, Belgium

**Keywords:** Motor control, Ageing

## Abstract

Mediolateral weight-shifting is an important aspect of postural control. As it is currently unknown whether a short training session of mediolateral weight-shifting in a virtual reality (VR) environment can improve weight-shifting, we investigated this question and also probed the impact of practice on brain activity. Forty healthy older adults were randomly allocated to a training (EXP, n = 20, age = 70.80 (65–77), 9 females) or a control group (CTR, n = 20, age = 71.65 (65–82), 10 females). The EXP performed a 25-min weight-shift training in a VR-game, whereas the CTR rested for the same period. Weight-shifting speed in both single- (ST) and dual-task (DT) conditions was determined before, directly after, and 24 h after intervention. Functional Near-Infrared Spectroscopy (fNIRS) assessed the oxygenated hemoglobin (HbO_2_) levels in five cortical regions of interest. Weight-shifting in both ST and DT conditions improved in EXP but not in CTR, and these gains were retained after 24 h. Effects transferred to wider limits of stability post-training in EXP versus CTR. HbO_2_ levels in the left supplementary motor area were significantly increased directly after training in EXP during ST (change < SEM), and in the left somatosensory cortex during DT (change > SEM). We interpret these changes in the motor coordination and sensorimotor integration areas of the cortex as possibly learning-related.

## Introduction

Weight-shifting constitutes an important component of dynamic postural control, which refers to the ability to keep one’s bodyweight within the base of support^1^. With age, weight-shifting becomes slower^2^ and less accurate^1^. Weight-shifting deficits, especially in the mediolateral direction^3,4^, were found to account for 41% of falls in older adults in one study^5^. Age-related increases in fall risk go hand in hand with an increased risk of fall-related injuries^6^, loss of mobility and mortality^7^. Therefore, strategies to ameliorate these problems are urgently needed. The few studies that investigated the effects of weight-shift training showed promising results^8,9^. Virtual reality (VR) programs have the potential to support such training by increasing the challenge and motivation of practice, as well as by providing direct feedback^10^. Currently, however, it is unclear whether improvements due to VR-based weight-shift training are retained, resist interference from dual-task (DT) demands, and generalize to improvements in balance performance beyond the training context. Even less is known about the neural correlates of training-induced weight-shift changes.

Most studies on learning-induced neural activation changes have focused on upper extremity tasks^11–14^, revealing a shift from anterior to posterior brain regions, suggesting a decreased dependence on attentional mechanisms with learning. Even though training-induced neural activation changes seems to be comparable between young and older adults^15^, motor learning is affected by age-related reorganizational changes in the brain^12^. During sequential finger movements, more brain activity is needed and more areas are recruited to reach comparable levels of performance in older versus young adults^16^.

Functional Near-Infrared Spectroscopy (fNIRS) is a mobile neuroimaging method able to capture brain hemodynamics when in the upright position, albeit at a much lower spatial resolution than fMRI^17^. fNIRS has already been widely used to study the underlying mechanisms of balance control, as described in a recent review^18^. In a previous study, we investigated the age-related brain activation pattern during a mediolateral VR weight-shifting task in older and young adults, showing increased brain activity with age^2^, particularly in the prefrontal cortex (PFC), supplementary motor area (SMA) and somatosensory cortex (SSC). Only three studies investigated training-induced neural activation changes with fNIRS, assessing the PFC^19^, motor cortices^20^ and sensorimotor area^21^. Importantly, these studies were done in young adults and athletes, and used different timelines and tasks. The first study found that 20 h of balance training within a dance video game resulted in a decrease in PFC activation^19^. The other studies showed that activations in the SMA^20^, primary motor cortex (M1), and parietal cortex^21^ were increased after 5 short trials of training on a tilting platform^20^ and 2 sessions of multimodal balance training^21^. These findings documented that learning-related changes in the brain could be detected with fNIRS after short training periods. It is yet unclear if and how weight-shift training influences the neural activity in older adults.

Given the above gaps in the literature, we set out to investigate older adults’ ability to improve weight-shifting using a VR-game and whether these effects would be retained after a period without training. Additionally, we wanted to examine whether the effects of weight-shift training were maintained when cognitively distracted^22^. As weight-shifting is important for maintaining stability during the majority of posture and gait tasks^23^, this study also assessed transfer to general balance performance and limits of stability outside the game setting. Finally, we aimed to elucidate the training-related neural activation changes of VR-based weight-shift training using fNIRS. Based on earlier work, we expected that older adults would be able to improve weight-shifting, balance performance and limits of stability after a single training session^8^ and that these changes would be maintained over 24 h^24^. We were uncertain about whether such improvements would hold when exposed to DT distraction^25^. We also expected that training-induced neural activation changes would be apparent mostly in the hemodynamics of the frontal and motor areas of the brain, in line with previous studies^19–21^. More specifically, as we previously found the PFC to be highly involved in our VR weight-shifting task in older adults^2^, we expected a rise in cortical HbO_2_ levels directly after training and a lowering at retention indicating an increase of neural efficiency^11,14,18^.

## Results

Some missing data were apparent due to recording problems (once due to the fNIRS-cap and once due to the wasp game). One participant in EXP was not willing to perform the DT condition, and one participant in CTR found the fNIRS system uncomfortable at retention.

### Participant characteristics

Participants were recruited, trained and tested between January 4th and June 16th, 2021. Forty-three healthy older adults were initially recruited for participation, as three participants had to be excluded after signing the informed consent. Two did not meet the in/exclusion criteria (one had a MoCA score below 26 and one was younger than 65 years). The third had a large difference in leg length (2.5 cm), influencing weight-shifting performance. Table 1 shows the clinical characteristics of the included 40 healthy older adults, all of whom performed the trial as planned. Groups showed no difference on all demographic and cognitive tests. In addition, no differences in baseline balance performance, as assessed by the MiniBEST, and baseline sleep quality measures were found. Most participants reported no falls in the past 6 months, with only one participant in EXP reporting two falls and three participants in CTR reporting one fall.

**Table 1:** Participant characteristics for the training and control group.

	EXP (N = 20)	CTR (N = 20)	p-value
Age (years)	71.00 (67.00, 74.00)	71.00 (67.25, 75.00)	0.529
Height (cm)	1.69 ± 0.09	1.69 ± 0.09	0.619
Weight (kg)	70.80 ± 11.79	71.17 ± 11.80	0.500
SARC-F (0–10)	0.00 (0.00, 1.00)	0.50 (0.00, 1.00)	0.221
FES-I (16–64)	17.00 (16.25, 20.50)	19.00 (17.00, 23.50)	0.174
miniBEST (0–28)	25.80 ± 1.64	24.95 ± 1.88	0.858
#Falls last 6 months	0.00 (0.00, 0.00)	0.00 (0.00, 0.00)	0.128^a,b^
PSQI (0–21)	4.10 ± 2.67	4.05 ± 2.14	0.197
Quality of sleep (0–3)	1.00 (0.00, 1.00)	1.00 (0.00, 1.00)	0.592^a^
Quantity of sleep (h)	7.29 ± 1.29	7.15 ± 1.01	0.646
MoCA (0–30)	27.50 (26.25, 29.00)	28.00 (26.25, 29.75)	0.583

Normally distributed data are displayed as mean ± SD, and not normally distributed data as median (first quartile, third quartile). *EXP* training group, *CTR* control group, *SARC-F* Sarcopenia Questionnaire, *FES-I* Falls Efficacy Scale International, *MoCa* Montreal Cognitive Assessment. ^a^X^2^. ^b^One participant in EXP reported 2 falls and 3 participants in CTR reported 1 fall.

### Behavioral results

#### Training effects on weight-shifting performance in ST

**Figure 1:**
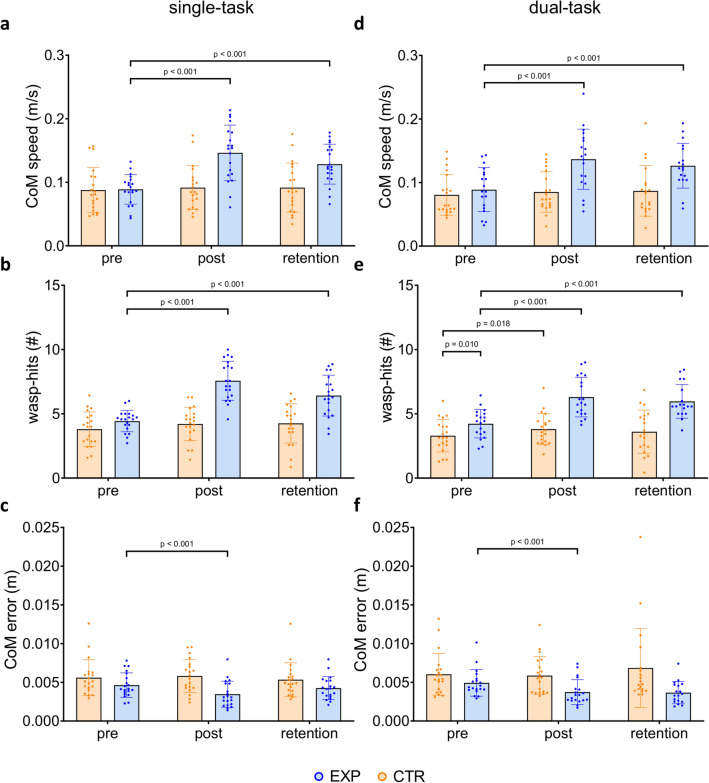
(**a**,**d**) Center of mass (CoM) speed (m/s); (**b**,**e**) wasp-hits (#); and (**c**,**f**) CoM error (m) during the wasp game single- and dual-task during pre-, post- and retention assessments for the experimental (EXP) and control (CTR) group.

Significant time by group interactions were revealed for all three outcomes (speed: F_(2,38)_ = 15.53, p < 0.001, Fig. 1a; wasps: F_(2,38)_ = 30.20, p < 0.001, Fig. 1b; error: F_(2,37)_ = 7.20, p = 0.002, Fig. 1c). Subsequent post-hoc testing showed that during baseline, both EXP and CTR showed no difference in weight-shifting speed (EXP: 0.09 ± 0.02 m/s, CTR: 0.09 ± 0.04 m/s), trajectory error (EXP: 0.005 ± 0.002 m, CTR: 0.006 ± 0.002 m), and the amount of wasps hit (EXP: 4.43 ± 0.83 wasps, CTR: 3.81 ± 1.34 wasps, trial-averages). Directly after training, EXP could increase their weight-shifting speed to 0.15 ± 0.04 m/s (d = 1.33) and the wasp-hits to 7.47 ± 1.42 (d = 1.76). At 24 h retention, these effects were largely maintained as weight-shifting speed was 0.13 ± 0.03 m/s (d = 0.94) and wasp-hits amounted to 6.42 ± 1.59 (d = 1.14). Moreover, trajectory error decreased in EXP directly after training (0.003 ± 0.002, d = − 0.61) but this decrease was not seen at 24 h-retention (0.004 ± 0.001 m). In the absence of training, speed (post: 0.09 ± 0.03 m/s, retention: 0.09 ± 0.04 m/s), wasp-hits (post: 4.21 ± 1.30 wasps, retention: 4.26 ± 1.53 wasps) and error (post: 0.006 ± 0.002 m, retention: 0.005 ± 0.002 m) did not change in CTR. Most importantly, EXP revealed large effect sizes for the primary outcome weight-shifting speed (pre-post: d = 1.33, pre-retention: d = 0.94).

#### Training effects on weight-shifting performance in DT

When adding the cognitive DT to the weight-shifting assessments, a comparable pattern of interaction effects was found (speed: F_(2,35)_ = 16.56, p < 0.001; Fig. 1d, wasps: F_(2,37)_ = 23.01, p < 0.001; Fig. 1e, error: F_(2,36)_ = 7.83, p = 0.001; Fig. 1f), with similar effect sizes for weight-shifting speed (pre-post: d = 1.40, pre-retention: d = 1.03). At baseline, both groups showed comparable weight-shifting speed (EXP: 0.09 ± 0.04 m/s, CTR: 0.08 ± 0.03 m/s) and error (EXP: 0.005 ± 0.002 m, CTR: 0.006 ± 0.003 m). However, participants in EXP already hit significantly more wasps compared to CTR (EXP: 4.23 ± 1.10 wasps, CTR: 3.30 ± 1.26 wasps) at baseline. After training, increases in weight-shifting speed (0.14 ± 0.05 m/s, d = 1.40) and wasp-hits (6.30 ± 1.54, d = 1.88) were found in EXP. After 24 h these improvements were retained (speed: 0.13 ± 0.04 m/s, d = 1.03; wasps: 5.97 ± 1.31, d = 1.24). Trajectory error decreased in EXP directly after training (0.004 ± 0.002 m, d = − 0.78) but this decrease was not seen at 24 h-retention (0.004 ± 0.001 m). Similar to ST, participants in CTR did not show an improvement in DT weight-shifting speed (post: 0.09 ± 0.03, retention: 0.09 ± 0.04) and trajectory error (post: 0.006 ± 0.002 m, retention: 0.007 ± 0.005 m). However, DT wasp-hits increased to 3.81 ± 1.22 (d = 0.45) from pre- to post-assessment in CTR, although this improvement was not significant anymore at 24 h-retention (3.61 ± 1.68 wasps). When checking the task-dependency of learning effects, the three-way group*time*task interaction was not significant, indicating no difference in learning gains on weight-shifting speed in ST and DT.

#### Training effects on functional limits of stability and overall balance

**Figure 2:**
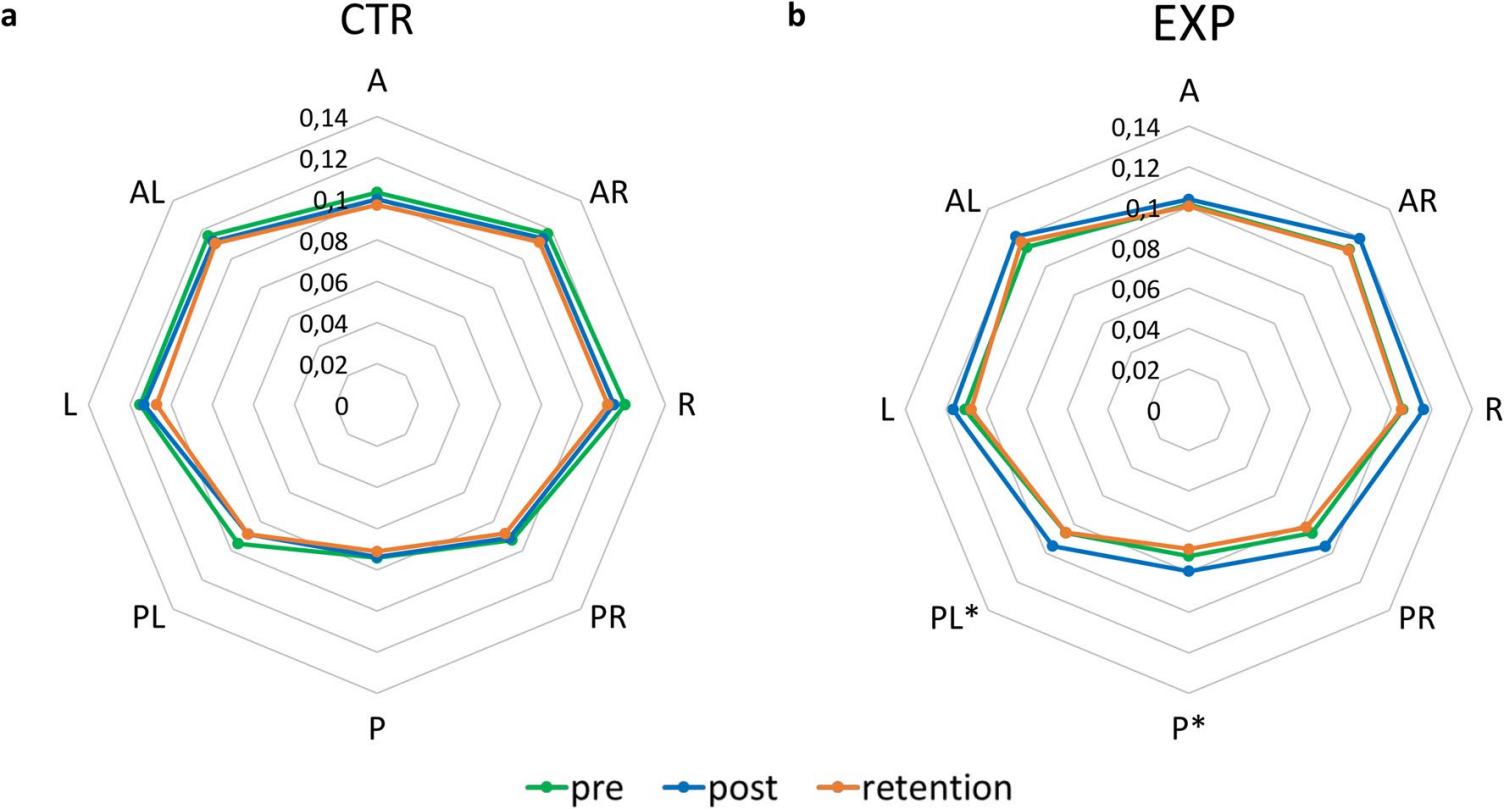
Functional limits of stability (fLOS) for the (**a**) control and (**b**) training group during pre-, post- and retention assessments. *A* anterior, *AR* anterior-right, *R* right, *PR* posterior-right, *P* posterior, *PL* posterior-left, *L* left, *AL* anterior-left. *Significant group*time interaction effect (p < 0.05).

Figure 2a,b show the radar plots of the functional limits of stability (fLOS) in the two groups before and after training, outside the game environment. There were two significant time*group interaction effects, showing increased tendencies to move further to the posterior (F_(2,38)_ = 4.11, p = 0.024) and posterior-left (F_(2,37)_ = 6.55, p = 0.004) directions in EXP compared to CTR. For these directions, the gains were found from pre- to post-training (posterior: 0.072 ± 0.014 m to 0.080 ± 0.015 m; posterior-left: 0.086 ± 0.017 m to 0.095 ± 0.019 m). Of note, these changes were not significant at 24 h-retention. Additionally, the CTR had larger fLOS to the right compared to the EXP at baseline (CTR: 0.121 ± 0.021 m, EXP: 0.106 ± 0.023 m, p = 0.026). Interestingly, the three-way group*time*direction interaction was not significant, confirming that transfer effects to stability limits were absent in the majority of directions (6 out of 8). No transfer to overall balance capacity was revealed on the MiniBEST scores across time points for both EXP (pre: 25.80 ± 1.64, retention: 25.80 ± 1.47) and CTR (pre: 24.95 ± 1.88, retention: 25.10 ± 1.77).

### fNIRS results

#### Training effects on cortical hemodynamics

**Figure 3:**
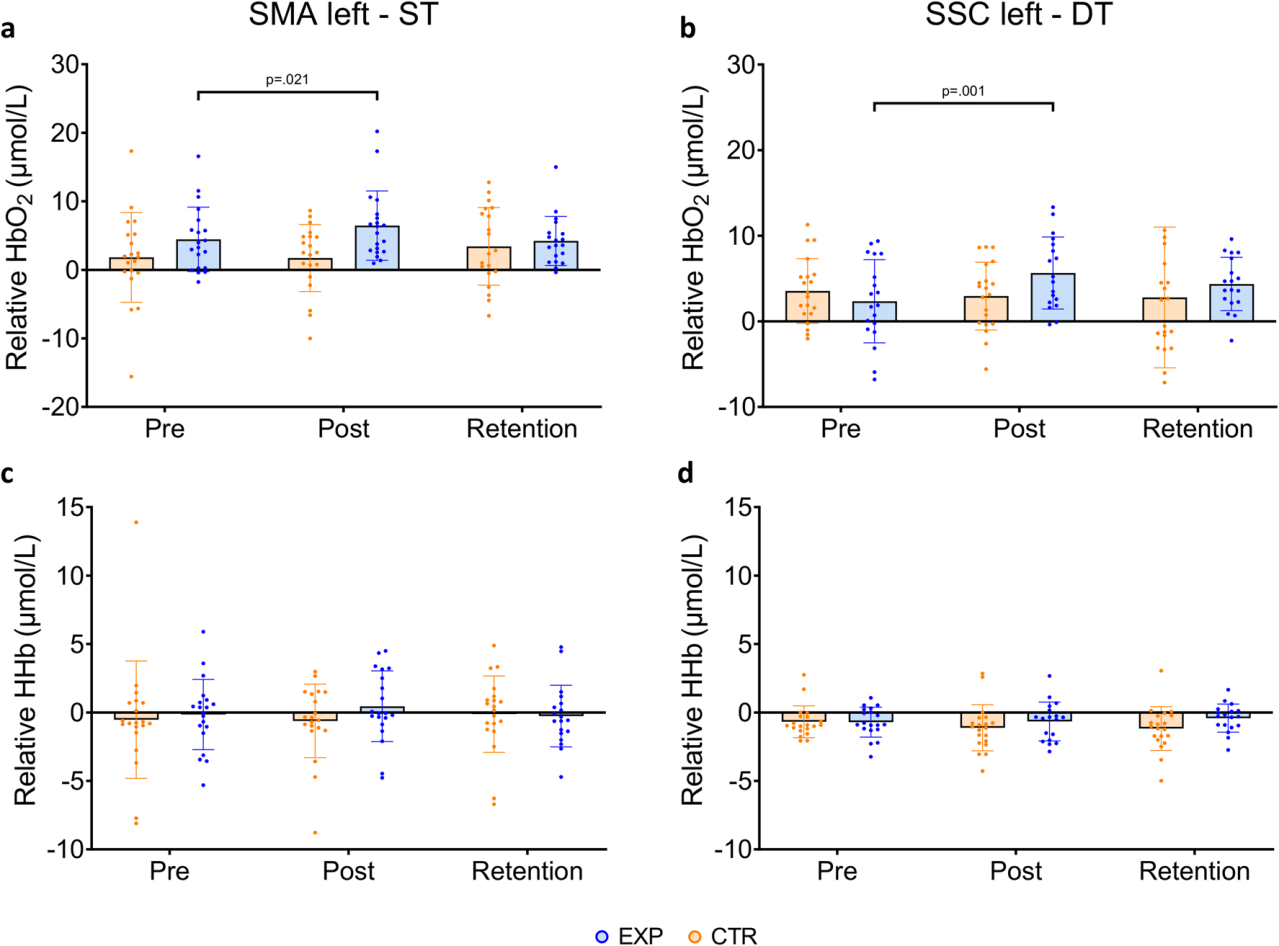
(**a**) Relative HbO_2_ levels for the SMA left during ST, and (**b**) SSC left during DT, during pre-, post- and retention assessments for the EXP and CTR. (**c**) Relative HHb levels for the SMA left during ST, and (**d**) SSC left during DT, during pre-, post- and retention assessments for the EXP and CTR. Significant within-group post-hoc tests are displayed here. Note that one outlier at retention fell outside of the graphs’ range (SSC left HbO_2_ level = 30.16 μmol/L), to make the spread of other data points more visible. *FEF* frontal eye fields, *SSC* somatosensory cortex, *HbO*_*2*_ oxygenated hemoglobin, *ST* single-task, *DT* dual-task, *EXP* training group, *CTR* control group.

Cortical hemodynamics during ST showed a significant interaction effect for the SMA left (F_(2,38)_ = 3.58, p = 0.038; see Fig. 3a). Within-group post-hoc testing revealed higher HbO_2_ levels in EXP during post- compared to pre-training. (t_(45)_ = 2.67, p = 0.010) with an effect size of d = 0.40 indicating a moderate effect. During DT conditions, the SSC left showed a significant interaction effect (F_(2,36)_ = 5.70, p = 0.007; see Fig. 3b). Post-hoc testing indicated a similar pattern as during ST, with higher HbO_2_ levels in EXP directly after training versus baseline (t_(42)_ = 3.64, p = 0.001, d = 0.56). There were no significant group by time interaction effects for the other ROIs. When examining whether the DT impacted on the overall result pattern, it was found that the three-way group*time*task interaction was non-significant for all ROIs. Overall, effect sizes between-time points seemed to be higher in EXP compared to CTR (see Table 2). In addition, no significant effects were found for HHb changes (see Fig. 3c,d and Supplementary Table 1).

**Table 2:** fNIRS HbO_2_ levels and interaction effects during wasp game ST and DT.

	EXP (N = 20)				CTR (N = 20)				Group*time interaction
	Pre	Post	Retention	Effect sizes^a^	Pre	Post	Retention	Effect sizes^a^	
ST									
PFC left	1.16 ± 4.23	1.81 ± 5.54	0.60 ± 5.59	0.06, − 0.13	2.39 ± 10.47	1.12 ± 7.61	1.94 ± 6.58	− 0.14, − 0.02	0.446
PFC right	1.90 ± 3.80	3.45 ± 8.12	3.34 ± 6.08	0.24, 0.14	1.17 ± 7.78	1.94 ± 6.48	3.90 ± 6.78	0.11, 0.22	0.730
FEF left	2.42 ± 4.46	2.85 ± 5.68	1.19 ± 4.06	0.10, − 0.14	1.40 ± 4.47	1.77 ± 4.16	0.72 ± 4.65	0.03, − 0.15	0.950
FEF right	1.66 ± 2.19	1.44 ± 5.43	2.43 ± 5.64	− 0.02, 0.11	0.90 ± 5.98	1.63 ± 4.13	2.45 ± 4.02	0.09, 0.19	0.848
SMA left	**4.46 ± 4.70**^**†**^	**6.46 ± 5.06**^**†**^	4.22 ± 3.59	**0.40**, 0.08	1.82 ± 6.55	1.73 ± 4.90	3.43 ± 5.67	− 0.10, 0.06	**0.038**
SMA right	4.31 ± 4.06	6.83 ± 7.17	5.49 ± 6.92	0.34, 0.16	2.48 ± 5.97	1.11 ± 6.72	2.98 ± 6.91	− 0.20, − 0.02	0.064
PMC left	3.00 ± 3.56	4.58 ± 4.26	3.37 ± 4.97	0.21, − 0.01	4.16 ± 7.29	3.62 ± 5.73	5.11 ± 6.35	− 0.05, 0.13	0.178
PMC right	2.70 ± 3.51	4.24 ± 5.69	6.28 ± 8.65	0.23, 0.28	4.00 ± 5.69	3.75 ± 5.29	5.00 ± 5.53	− 0.02, 0.14	0.471
SSC left	2.20 ± 3.58	4.62 ± 3.45	4.61 ± 4.12	0.37, 0.29	2.98 ± 4.07	2.37 ± 3.85	3.29 ± 5.46	− 0.07, 0.07	0.055
SSC right	3.99 ± 3.68	4.71 ± 5.14	4.95 ± 4.10	0.13, 0.15	4.09 ± 5.41	2.43 ± 5.28	3.12 ± 5.34	− 0.30, − 0.16	0.095
DT									
PFC left	− 0.08 ± 4.61	2.47 ± 6.03	0.94 ± 5.59	0.21, 0.06	1.43 ± 5.99	4.19 ± 7.70	1.62 ± 8.88	0.33, 0.05	0.865
PFC right	0.48 ± 4.68	3.09 ± 8.45	1.11 ± 7.55	0.32, 0.03	1.08 ± 8.60	1.03 ± 9.58	1.66 ± 5.34	− 0.01, 0.07	0.228
FEF left	2.19 ± 3.84	3.64 ± 4.74	0.77 ± 3.44	0.26, − 0.15	0.46 ± 5.16	1.71 ± 5.47	2.36 ± 4.71	0.21, 0.22	0.161
FEF right	2.34 ± 6.65	2.98 ± 5.72	3.14 ± 5.42	0.11, 0.14	0.70 ± 5.98	2.68 ± 4.97	1.02 ± 4.11	0.17, − 0.03	0.546
SMA left	5.63 ± 5.98	6.76 ± 5.33	5.06 ± 4.24	0.27, 0.09	1.43 ± 8.13	2.87 ± 4.68	5.50 ± 9.49	0.01, 0.19	0.210
SMA right	4.72 ± 5.34	6.52 ± 6.09	6.23 ± 5.64	0.26, 0.21	3.34 ± 7.29	3.72 ± 5.37	3.66 ± 4.87	0.01, − 0.01	0.258
PMC left	4.26 ± 5.42	5.70 ± 5.02	3.69 ± 4.76	0.15, − 0.07	5.93 ± 11.21	4.26 ± 8.87	5.18 ± 10.98	− 0.19, − 0.00	0.197
PMC right	1.50 ± 6.37	4.87 ± 5.30	6.99 ± 11.36	0.39, 0.35	4.16 ± 7.33	5.73 ± 8.57	4.71 ± 6.89	0.26, 0.10	0.451
SSC left	**2.34 ± 4.85**^**†**^	**5.65 ± 4.21**^**†**^	4.37 ± 3.12	**0.56**, 0.23	3.55 ± 3.78	2.95 ± 9.95	2.79 ± 8.23	− 0.06, − 0.06	**0.007**
SSC right	4.35 ± 6.01	4.68 ± 6.99	4.64 ± 6.09	0.07, 0.06	3.68 ± 6.60	3.91 ± 5.92	3.34 ± 6.34	0.00, − 0.06	0.791

Values are displayed as mean ± SD (μmol/L). Bold values indicate statistical significance and effect sizes of significant post-hoc tests. Note that only within-group time effects were explored post-hoc. ^**†**^Significant within-group time-effects (p < 0.05). ^a^Pre-post and pre-retention effect sizes based on t-values. *ST* single-task, *DT* dual-task, *EXP* training group, *CTR* control group.

### Exploratory analyses

To determine which people responded best to training, correlation analyses were conducted and revealed that participants in EXP who had a better overall balance at baseline (higher MiniBEST score) also tended to have a larger ST speed improvement from pre-assessment to 24 h-retention (r = 0.456, p = 0.043, see Supplementary Fig. 1). However, this correlation did not survive corrections for multiple comparisons. No other significant correlations were found.

**Figure 4:**
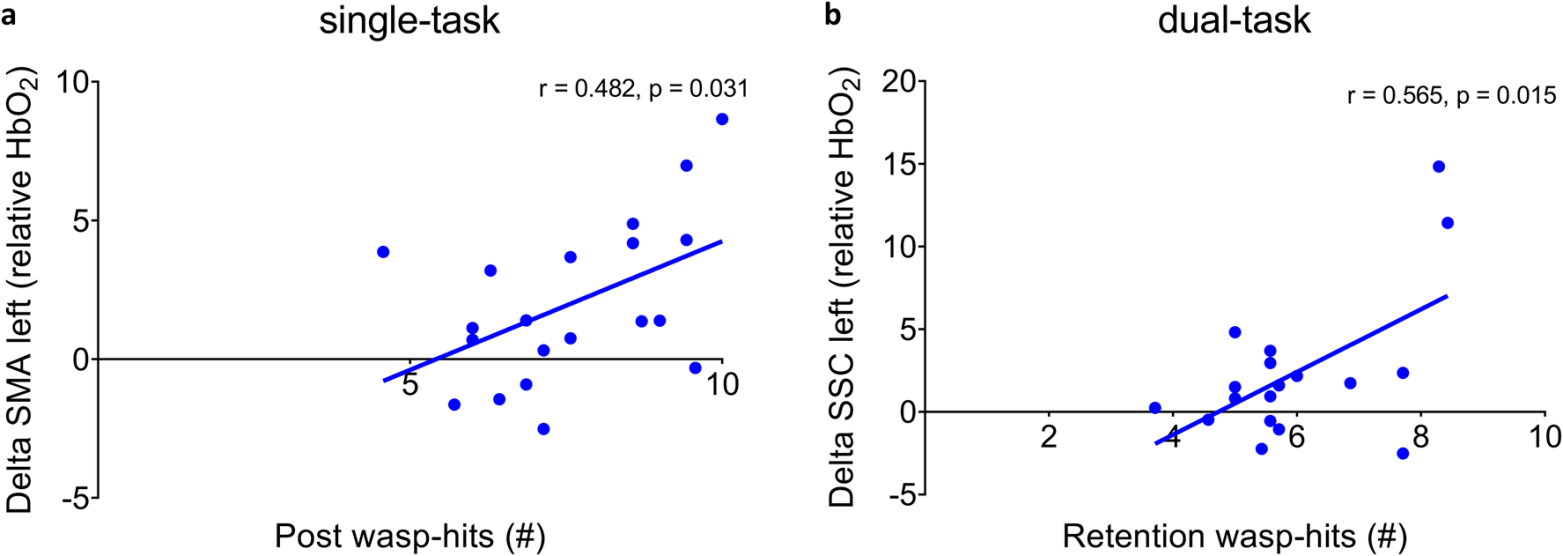
(**a**) Correlation between the change (delta) in relative SMA left HbO_2_ levels from pre- to post-assessment and wasp-hits at post-assessment during ST, and (**b**) correlation between the change (delta) in SSC left HbO2 levels from pre-assessment to 24 h-retention and wasp-hits at 24 h-retention during DT. *SMA* supplementary motor area, *SSC* somatosensory cortex, *ST* single-task, *DT* dual-task.

To explore the association of the HbO_2_ changes with behavioral changes, a second correlation analysis was conducted in EXP. We included the two brain regions and behavioral outcomes which showed significant group*time interactions (12 comparisons, see Supplementary Fig. 2 and Supplementary Fig. 3). Results showed that an increase in the left SMA HbO_2_ levels from pre- to post-training and in the left SSC HbO_2_ levels from pre-training to 24 h-retention were correlated with more wasp hits after training (SMA left: r = 0.482, p = 0.031; SSC left: r = 0.565, p = 0.015; see Fig. 4). Note, that none of these correlations survived correction for multiple comparisons, and displayed no significant correlations with weight-shifting speed and error.

## Discussion

This is the first study that investigated whether a single-session VR-based balance training led to changes in weight-shifting behavior and brain activity in older adults. Older adults were able to improve their weight-shifting capacity after the session during ST and DT test-conditions, the latter reflecting that the gains were resistant to DT-interference. In addition, and as hypothesized, training effects were retained after 24 h and transferred to improved limits of stability outside the game-setting, though this transfer was only present in two directions and disappeared the next day. We also hypothesized that training-induced changes in HbO_2_ levels in the frontal and motor areas would underlie learning results. This assumption was partly confirmed, as HbO_2_ levels were increased at post- compared to pre-training in the left SMA during the ST-tests. Increases were also witnessed in the left SSC during DT-tests. These changes in brain hemodynamics tended to be associated with better game-performance after training. Together, these results suggest that older adults were able to improve weight-shifting and modulate task-related brain activity in regions involved in motor planning (SMA) and sensorimotor integration (SSC).

The finding that EXP displayed better weight-shifting compared to CTR was not only confirmed by better game outcomes (increases in wasp-hits), but more importantly by a better control of the CoM (higher speed and accuracy). Learning led to larger effect sizes in EXP (speed: d ≥ 0.94) versus CTR (speed: d ≤ 0.09), which were retained over a 24 h-retention period. These findings are in line with a systematic review on balance training in older adults^26^, and the effects sizes found after a 4-week^27^ and 5-week^28^ balance training program. Caljouw et al.^8^ also found improvements in target-directed weight-shifting performance after a single-session of training. They revealed that an implicit training method, in which less focus was directed towards task details, led to better results than explicit training in older adults, possibly due to its relative independence of working memory^29^. Although the wasp-game was not an implicit training mode^30^, the fact that attention was focused on the wasps rather than on the weight-shifts^31^ may partly explain why we could confirm large training effects after only one session.

Next, we could also demonstrate that weight-shift training effects were resilient to DT interference. Similar results were found in other studies investigating DT balance training^8,32^ showing that DT performance remained intact at follow-up^25^. Even though participants in the current study received ST training, as no deliberate secondary task was added to the practice environment, the finding that DT performance improved may be explained by the fact that the VR wasp game combined several task components in the motor-cognitive domain. This enriched training environment may have facilitated distraction-resistant learning^33^. Another explanation could be that the cognitive task applied during the DT-assessment was too easy and therefore did not evoke test-interference in this cohort. However, our previous study showed that adding a serial subtraction task incurred significant DT-cost during the wasp game in a different cohort of older adults^2^.

Importantly, after mediolateral weight-shift training the EXP showed improvements of the stability limits in two directions. These fLOS gains could be considered as indications of near-transfer, referring to the greater likelihood to generalize learning outcomes when a high degree of similarity between the learned and the transfer task is apparent^34,35^. However, this near-transfer was not found for the mediolateral direction, disappeared after 24 h and no far-transfer to overall balance performance (MiniBEST score) was found. In contrast, Hatzitaki et al.^9^ showed that balance improvements after a 4-week weight-shift training program were direction- and training-specific, especially in the anterior–posterior direction. Likewise, mediolateral and anterior–posterior weight-shift practice of a similar duration seemed to increase stability limits in the trained direction only^36^ in a cohort of older women. The fact that the current training study entailed only a single-session of 25 min in contrast to the 4-week training durations in previous studies^27,28^, might explain the discrepancy with former work.

As for neural activity, previous work demonstrated that changes in the brain are different for acquisition than retention^37^. Based on this work, we expected a general increase in cortical HbO_2_ levels for acquisition and a decrease at retention when the brain becomes more efficient with learning^11,14,18^. Indeed, we found an increase in HbO_2_ levels in the left SMA directly after training, although only during ST-assessment. Interestingly, these changes tended to be related to a larger number of wasp hits after training. Similar training-related increases in SMA-HbO_2_ levels were found directly after training on a balance platform in young adults^20^. The SMA signifies a region involved during postural control^38^, and more specifically in motor coordination and motor adaptation of imagined locomotion^39^. The increased SMA-HbO_2_ levels after the present weight-shift training suggest that an increased reliance on real time motor planning was necessary in older adults to achieve better wasp game performance^22,40^. Note, we recently found that the fNIRS test–retest reliability, when measuring cortical hemodynamics twice on the same day, was fair for the SMA left (ICC = 0.49, CI = [0.07, 0.77], SEM = 4.11 μmol/L). As the SMA-increases in HbO_2_ in this study did not exceed the earlier reported SEM-value (SMA-HbO_2_ change: 2.00 μmol/L)^41^, this finding needs to be interpreted with caution.

A similar pattern of results was found in the left SSC during DT-assessment, revealing an increase in HbO_2_ levels immediately after training. As a good reliability was found previously for the left SSC (ICC = 0.60, CI = [0.23,0.82], SEM = 2.51) and the HbO_2_ level increase exceeded the SEM (SSC-HbO_2_ change = 3.31 μmol/L)^41^, this result can be interpreted as robust. Here, the change from baseline to 24 h-retention tended to be correlated to more wasp-hits after the follow-up period. Therefore, we cautiously suggest that VR-based weight-shift training incurred higher brain activity in sensory integration regions for the purpose of motor programming^42^ which would be in agreement with previous results regarding a multimodal balance training^21^. Adding the visually cued serial subtraction task likely made the weight-shifting assessment more challenging in terms of sensory integration, explaining why the increase in SSC-HbO_2_ levels was found during DT- and not during ST-conditions.

Even though we expected that early learning from pre- to post-training would require an increasing need for attention^11,14,18^, no training-related hemodynamic changes were found for the PFC. Visually, the temporal changes of PFC-HbO_2_ levels in EXP seemed to suggest the expected pattern (see Table 2), but changes were not large enough and/or were too variable for reaching statistical significance. Earlier work on the same task revealed that older adults showed an elevated cortical activation, particularly in the PFC, compared to young adults during ST wasp game performance^2^. Yet, lower PFC-HbO_2_ levels were found during DT in the same older group, possibly due to limited attentional capacity^2^. In this training study, the lack of PFC changes may have been due to the high engagement of the PFC needed during the game, leaving no room to detect significant changes over time. In contrast, a multi-session dance video game showed a decrease in PFC activation as learning progressed^19^. The longer training period (20 h) might have been responsible for this decreased reliance on cognitive resources.

Earlier work also showed that learning is often accompanied by a shift of activity to subcortical regions^14,30,43^, which was not possible to investigate here due to the limitations of the fNIRS-technique. The fact that we did not find a decrease in overall cortical hemodynamics at retention may be due to our earlier finding that test–retest reliability was compromised after removing the cap^41^ and thus may have created variability. In addition, the fact that the HbO_2_ findings were not supported by changes in HHb might be explained by the lower signal to noise ratio. Changes in HHb were possibly overshadowed by both central and local physiological changes in response to the whole-body weight-shifting task, the latter of which are not picked up by the short-separation channels^44^.

This study represented a randomized controlled trial, whereby the tester was not blind to group allocation and the contrast included a passive control group. However, outcomes were based on objective measures. Due to an error in the a-priori sample size calculation, this study was probably underpowered for some outcomes. However, effect sizes of the significant results were higher than the sensitivity power analysis-derived threshold. In addition, the modest reliability of repeated fNIRS measures in some regions hampered a strong interpretation of the results. To optimize the validity of the fNIRS data, we incorporated short-separation channels and accelerometers into the setup and analysis, filtering out movement artefacts and systemic noise as stipulated by recent guidelines^45,46^. Future work is needed to clarify how the neural signals can be further distinguished from regional blood flow changes. The positive training effects, even after a short session, point to the need for a single-blind randomized controlled trial, including multiple training sessions, an active control group and a longer retention period, to investigate whether this type of intervention can improve balance control on transfer tasks and as such prevent falls in the older population. As most participants did not experience a fall in the last 6-months prior to testing, it is imperative to include fall-prone older adults in future studies.

However, the fact that weight-shifting capacity in older adults was improved with a single session of training is promising and supports the incorporation of weight-shifting VR-games in fall-prevention programs. Another important finding for the clinical field was that the results were also present in conditions with cognitive distraction, implying robust learning results. Future research is needed to investigate whether enriched weight-shift training can induce sustained neural changes and lead to transfer effects to overall balance capacity in older adults.

This study showed that a single training session improved mediolateral weight-shifting capacity in healthy older adults during both ST and DT assessments when compared to a control group without training. The gains were maintained after 24 h and showed short-term transfer to improvement of the limits of stability, although not in the mediolateral direction. The increased HbO_2_ levels in the brain after training in motor planning (SMA, change < SEM) and sensory-motor integration (SSC, change > SEM) regions underscore the potential for neuroplastic changes in older individuals.

## Materials and methods

### Participants

In total, we recruited forty-three healthy older adults through our local GDPR-proof databases, of which forty were included in the actual study protocol. Participants had to be able to stand upright for a minimum of 5 min without using an aid and were excluded if they presented any medical problems that could influence their performance (e.g. neurological disorders, balance impairments, chronic musculoskeletal problems, cardiovascular or respiratory disease, uncorrected vision, or diabetes). Participants with a Montreal Cognitive Assessment (MoCA) score lower than 26 were excluded. Study information was given before signing the informed consent in accordance with the declaration of Helsinki. Ethical approval was provided by the Ethics Committee Research UZ/KU Leuven (EC Research; S26917; 26 May 2020).

### Experimental procedure and tasks

#### Procedure

This randomized controlled trial was pre-registered at clinicaltrials.gov (ID: NCT04594148, first registration: 20/10/2020, including full trial protocol) and consisted of two test sessions at the Movement and Analysis Laboratory Leuven (MALL). Healthy older adults were randomly allocated to either the training (EXP, n = 20) or control group (CTR, n = 20) in a parallel design. Blocked computer-generated randomization was performed by an independent researcher in blocks of four and stratified for gender. The randomization sequence was revealed to the researcher after participant enrollment. On day 1, the EXP received a 25-min weight-shift training intervention within the VR wasp game (see section “Interventions”). Weight-shifting performance and cortical hemodynamics were assessed before (pre) and directly after training (post) with and without a cognitive DT. The next day (~ 24 h), participants in EXP would return to the lab and the same measurements were performed (retention) (see Supplementary Fig. 4). The CTR underwent the same procedures, except that they rested for the same duration as the training period. Weight-shifting speed was used as the primary outcome measure. Both groups were tested for fatigue by the visual analogue scale (VAS) directly before each assessment.

#### Interventions

Mediolateral weight-shift training was performed via a VR wasp game (see Supplementary Fig. 5b)^47^ described previously^2^. In short, participants had to shoot virtual wasps that appeared in alternating fashion on the left and right side of the screen, with a VR water jet. To activate the water jet, they had to shift their center of mass (CoM) beyond 80% of their individual functional limits of stability (fLOS; see Supplementary Fig. 5a), thereby providing a challenging and personalized training load. fLOS were recorded by shifting weight as far as possible in eight directions prior to game performance, and calculated as the mean of three trials for each direction separately. CoM position was calculated in real time within the D-Flow software (Motek Medical BV, Amsterdam, The Netherlands; version 3.28) and based on eight reflective markers placed on bilateral anatomical landmarks on the acromia, posterior superior iliac spines, lateral epicondyles, and lateral malleoli^48^, as recorded within the Nexus software (Vicon, Oxford Metrics, UK).

The 25-min training was divided into 10 blocks of 2.5 min with a 30 s rest period in between blocks. A longer break of 2 min was provided after five blocks with the opportunity to rest on a chair. Participants were instructed to hit as many wasps as possible, while maintaining their base of support and initial posture. To make the training more challenging, the wasps were randomly placed at positions higher or lower than the midline and were made bigger or smaller. As such, participants had to pay constant attention to the position and size of the wasp. Verbal feedback and encouragement was provided throughout the training. Participants in the CTR were asked to relax for 25 min, while seated.

#### Weight-shifting assessment

Weight-shifting assessments were performed at pre, post and retention. For this purpose, the wasp game was executed in both single- (ST) and dual-task (DT) conditions. During DT, participants had to perform serial subtractions in threes, while hitting the wasps (see Supplementary Fig. 5c), as previously described^2^. In contrast to the weight-shift training, the wasps were always placed on the horizontal midline and displayed in one size. Wasp game ST and DT assessments were performed in a fixed order and blocked design to allow for simultaneous fNIRS^49^ measurements. Blocks consisted of seven trials starting with 20 s of quiet stance while watching a pre-recorded video of the wasp game. This was alternated with 20 s of weight-shifting within the actual wasp game. Prior pilot testing revealed the highest true positive rate when using this specific block design. The fLOS were also determined as a general measure of balance capacity before and after training or rest as well as at 24 h-retention. The Mini Balance Evaluation Systems Test (MiniBEST)^49^ was administered before the intervention on day 1 and again on day 2 as a secondary outcome of general balance performance.

#### fNIRS assessment

Brain hemodynamics were captured with the continuous wave, single-phase NIRSport2 system (NIRx, Berlin, Germany), with LED wavelengths of 760 and 850 nm and a sampling frequency of 7.81 Hz, following consensus guidelines^50^. fNIRS data were recorded withinthe Aurora software (version 2020.7). Prior to data capture, cap size was chosen out of four possible sizes based on participants’ head circumference and positioned carefully on the head after determining the Cz position using the nasion, inion and pre-auricular anatomical landmarks. The Cz, CP2, and FC1 positions were marked on the head to standardize cap placement between day 1 and day 2. The fNIRS Optode Location Decider (fOLD) toolbox was used to determine source and detector locations covering five regions of interest (ROIs): the prefrontal cortex (PFC; Brodmann areas 9, 10 and 46), frontal eye fields (FEF; Brodmann area 8), premotor cortex (PMC; Brodmann area 6, lateral^51^), supplementary motor area (SMA, Brodmann area 6, medial^51^), and somatosensory cortex (SSC; Brodmann areas 1, 3, 5 and 7). These ROIs were chosen as they were found to be involved in performing the wasp task in our previous study^2^, capturing executive functioning (PFC)^52^, visual attention (FEF)^53^, motor planning (PMC)^54^, motor adaptation (SMA)^39^ and sensorimotor integration (SSC)^42^. In total, 16 sources and 16 detectors were used (~ 3 cm separation), of which two detectors served as a reference for 16 short-separation channels (~ 8 mm separation), one placed around each source. Two accelerometers were placed at the back of the head to enable control for excessive head movements. The whole fNIRS set-up was covered with a light-impermeable cap. After initial checks, good signal quality could be reached after the above-described cap preparation.

#### Cognitive assessment and other descriptors

After the experimental procedures on day 2, participants performed a descriptive test battery. We used the MoCA for assessment of cognitive ability. Other questionnaires included the Falls Efficacy Scale International (FES-I) to assess concern about falling, the sarcopenia questionnaire (SARC-F) and the Pittsburgh Sleep Quality Index (PSQI) capturing sleep quality and quantity over the last month and the night in between assessment day 1 and 2.

### Data processing

#### Weight-shifting data

Matlab 2018b (Mathworks, MA, USA) was used to process the CoM data as calculated in real time within the D-Flow software^48^. First, data were filtered using a low-pass filter (4th order Butterworth, 6 Hz cut-off). Second, the start and end of each weight-shift was delineated automatically. Weight-shifting was defined as the movement between the two 80% fLOS thresholds (in both directions), thereby excluding the weight-shift leading to the first wasp hit. Outcome measures were averaged within and over trials and included weight-shifting speed (primary outcome), weight-shifting accuracy (CoM trajectory error), the number of wasps hit and the fLOS in eight directions.

#### fNIRS data

**Figure 5:**
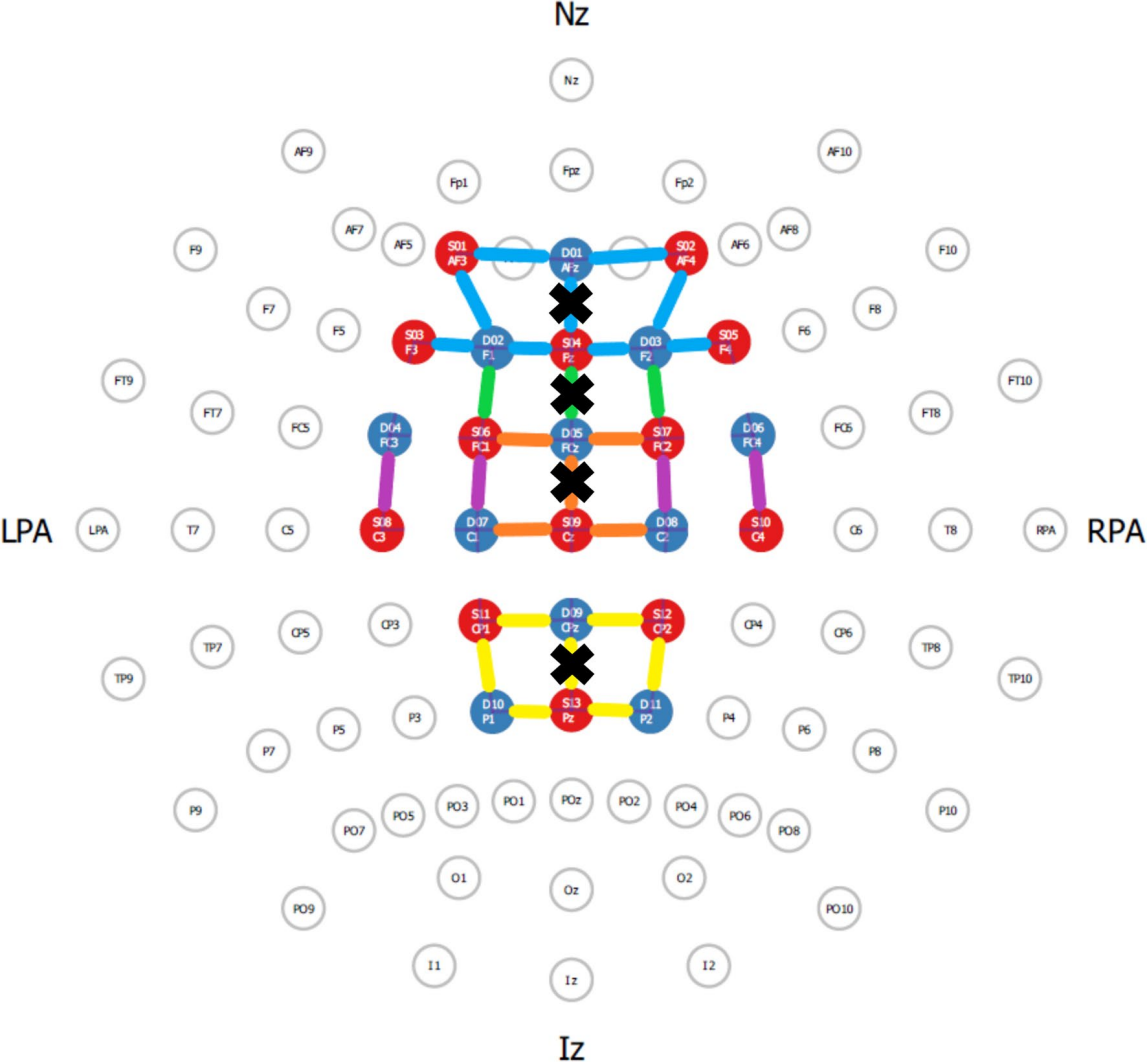
fNIRS lay-out with regions of interest (ROIs) according to fOLD toolbox, with a specificity level of 50%. The prefrontal cortex (PFC) is represented by the blue colored connections to the channels, the frontal eye fields (FEF) by the green channels, the supplementary motor area (SMA) by the orange channels, the premotor cortex (PMC) by the purple channels, and the somatosensory cortex (SSC) by the yellow channels. The channels situated at the midline were excluded (indicated by black crosses), and ROIs were analyzed for each hemisphere separately.

fNIRS data were imported into Matlab (version 2018b, Mathworks, MA, USA) and analyzed using the NIRS toolbox (open access; https://github.com/huppertt/nirs-toolbox)^55^. Processing steps included:

1. Visually checking raw signals and resampling signals to 5 Hz.
2. Converting raw signals into optical density using the Beer–Lambert law for which a partial path length correction factor of 0.1 was used.
3. Running a general linear model (GLM) with the autoregressive iteratively reweighted least squares (AR-IRLS) method^56^. To diminish the influence of movement artefacts and physiological noise, accelerometer and short-separation channel data were added as regressors^45,46^.
4. Averaging channels per hemisphere for each ROI, as determined within fOLD, and the human motor area template for separating the PMC and SMA^51^ (see Fig. 5). Channels were excluded if the spatial specificity was lower than 50%^57^ as well as when they traversed over the superior sagittal sinus, avoiding measuring cerebrospinal fluid instead of cortical hemodynamics^58^.
5. Calculating relative oxygenated hemoglobin (HbO_2_) and deoxygenated hemoglobin (HHb) (active trial − rest trial (μmol/L)) and averaging over trials.

As HbO_2_ levels have a higher signal-to-noise ratio compared to HHb^59,60^, HbO_2_ levels were analyzed as secondary outcomes measures, and HHb levels as tertiary ones. Prior to analysis, we investigated the test–retest reliability of HbO_2_ levels pre-post and pre-retention, the latter including cap removal^41^. Pre-post reliability for total ROIs ranged from fair (FEF, SMA) to excellent (PFC, PMC, SSC). However, pre-retention reliability became poor for the motor areas (SMA, PMC), and fair (PFC, SSC) to good (FEF) for the non-motor areas. We also captured the reliability for the hemispheres separately, which largely mirrored these results. The relevant reliability metrics, intraclass correlation coefficient (ICC) and standard error of measurement (SEM) were considered for the interpretation of significant findings.

### Statistical analysis

An a-priori sample size calculation (see clinicaltrials.gov, ID: NCT04594148) determined that we required 40 participants (20 per group). However, during data analyses, an error was found in that calculation, so an additional sensitivity power analysis was performed. From this, we determined that our sample of 40 is sufficient to detect effect sizes *d* ≥ 0.45 (alpha of 0.05, 80% power) in a repeated measures, within-between group interaction design.

SAS (Version 9.4 of the SAS System for Windows, SAS Institute Inc., Cary, NC, USA) and SPSS (IBM SPSS Statistics, Version 26) was used for data analysis, including all participants. Participant characteristics as well as behavioral and cortical outcomes at baseline were compared between groups with an independent *t*-test or Mann–Whitney *U* test, depending on data distribution. A constrained longitudinal data analysis (cLDA) approach^61^ was chosen to investigate the effect of a single-session weight-shift training on both behavioral and cortical outcomes across the three time points, based on the most optimal model parameters (Akaike’s information Criterion, AIC). Subject was used as random factor with an unstructured covariance matrix and time (pre, post, retention) as a repeated fixed factor. The interaction between EXP and CTL at post and retention compared to pre-training were dummy coded. Post-hoc Student’s *t*-tests were additionally performed for the within-group changes if interaction effects were significant. Age was added as a covariate, as currently there is no formula for adjusting the differential path lengths in older adults^50^. As fatigue, measured by the VAS, did not influence our primary and secondary outcome measures, this score was not included in the models. Previously, we investigated the effect of adding a cognitive task to the wasp game on cortical hemodynamics^2^. Therefore, separate models were used for ST and DT conditions in the current study. However, to investigate whether the DT effects were comparable to those obtained in ST, a three-way interaction between group, time and task was carried out in SPSS for the primary outcome only. A similar three-way interaction effect between group, time and direction was analyzed for the fLOS, to check the direction-specificity of significant group by time effects. Assumptions were checked prior to the analysis, including homoscedasticity, normality of residuals and linearity. Multiple testing was adjusted with the Bonferroni method, and p-values were set at 5% for significance. For primary and secondary outcomes, effect sizes based on t-values from the model were calculated to be able to interpret the clinical relevance of the findings^62^.

To explain the training effects, Pearson’s correlations between changes in ST weight-shifting speed (pre-post, pre-retention) and participant characteristics (age, MoCA, FES, SARC-F, PSQI, baseline MiniBEST) were calculated, resulting in 12 comparisons. As well, an exploratory correlational analysis (without correction for multiple comparisons) was carried out between changes in cortical hemodynamics for the ROIs that displayed significant interaction effects and weight-shifting performance.

### Supplementary Information

Supplementary Information.

## Data Availability

The datasets generated for this study will be made available upon reasonable request, which should be directed to the corresponding author.
