# Supplementary material for: Neural correlates of weight-shift training in older adults: a randomized controlled study
Source: Sci Rep. 2023 Nov 10;13:19609. doi: 10.1038/s41598-023-46645-4 (PMC10638445; doi:10.1038/s41598-023-46645-4)
Supplement: Supplementary file 1 — Supplementary Information. [file 41598_2023_46645_MOESM1_ESM.pdf]

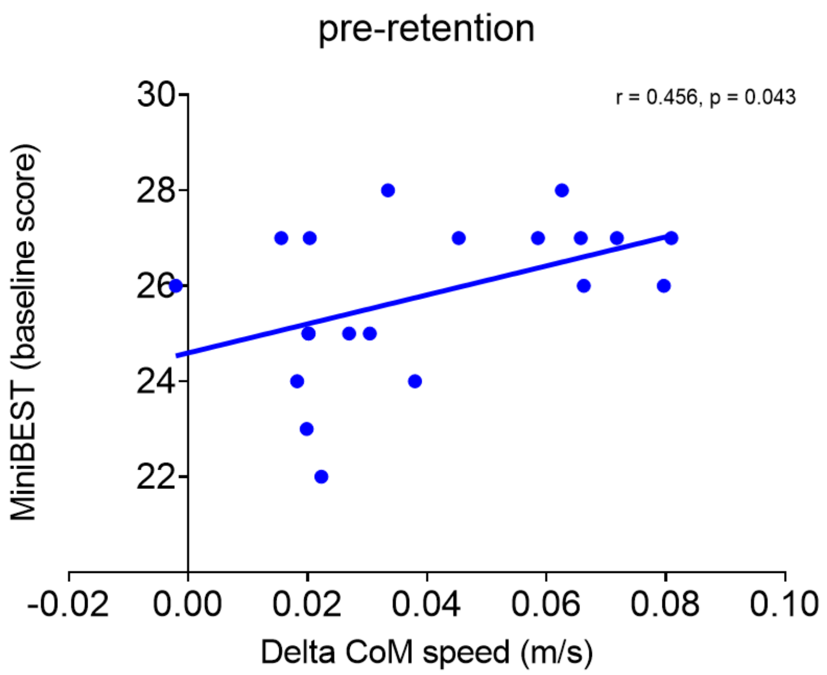


**Supplementary Figure 1.** Correlation between the baseline MiniBEST score and the delta (change) in CoM speed from baseline to 24h-retention during the wasp game single-task. CoM = center of mass; MiniBEST = minimental balance evaluation systems test.

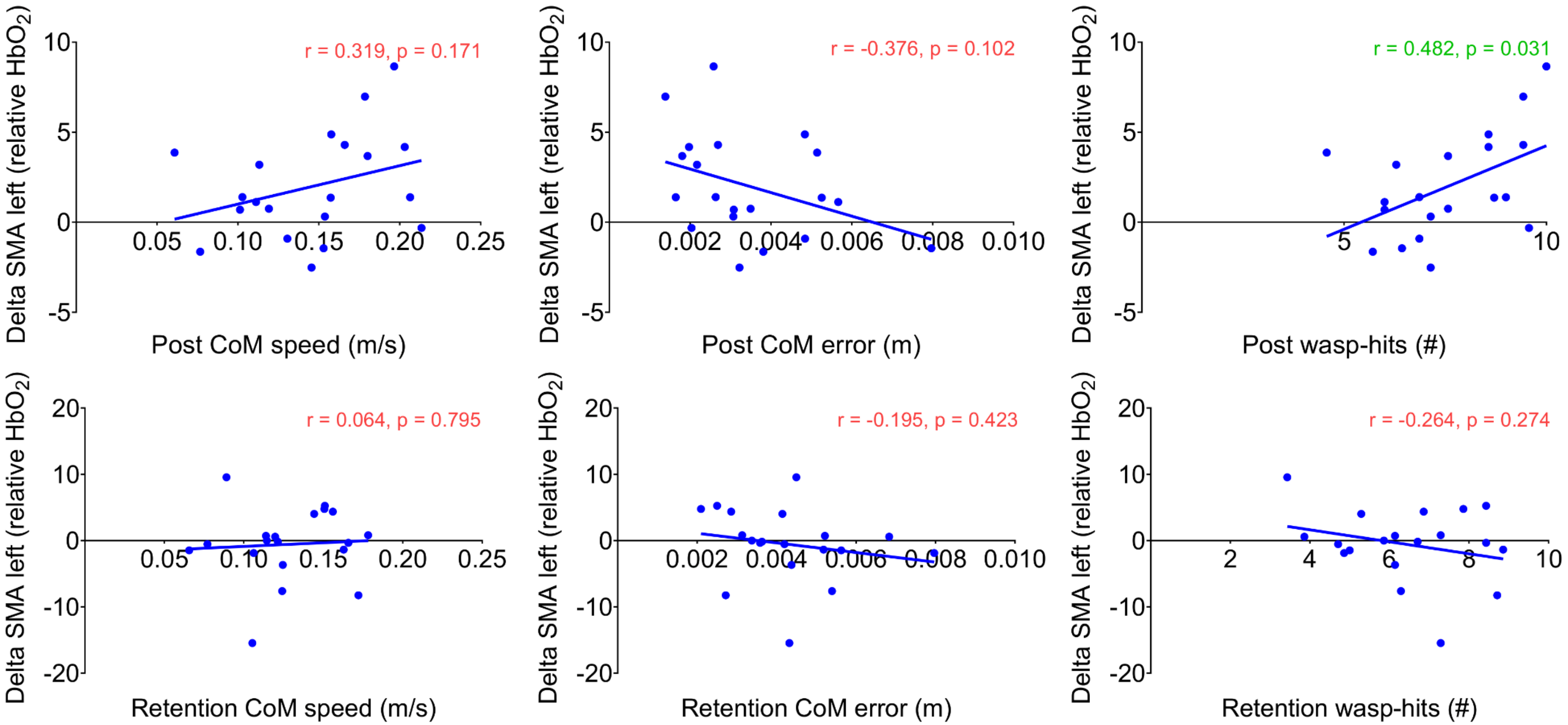


**Supplementary Figure 2.** Correlations between the delta (change) in relative SMA left $HbO_2$ levels from pre- to post-assessment and VR wasp game performance at post-assessment (CoM speed, CoM error, wasp-hits) (top panels), and between the delta (change) in relative SMA left $HbO_2$ levels from pre-assessment to 24h-retention and VR wasp game performance at 24h-retention (CoM speed, CoM error, wasp-hits) (bottom panels). All correlations were performed in the EXP and ST-condition only. CoM = center of mass; SMA = supplementary motor area; CoM = center of mass; EXP = training group; ST = dual-task.

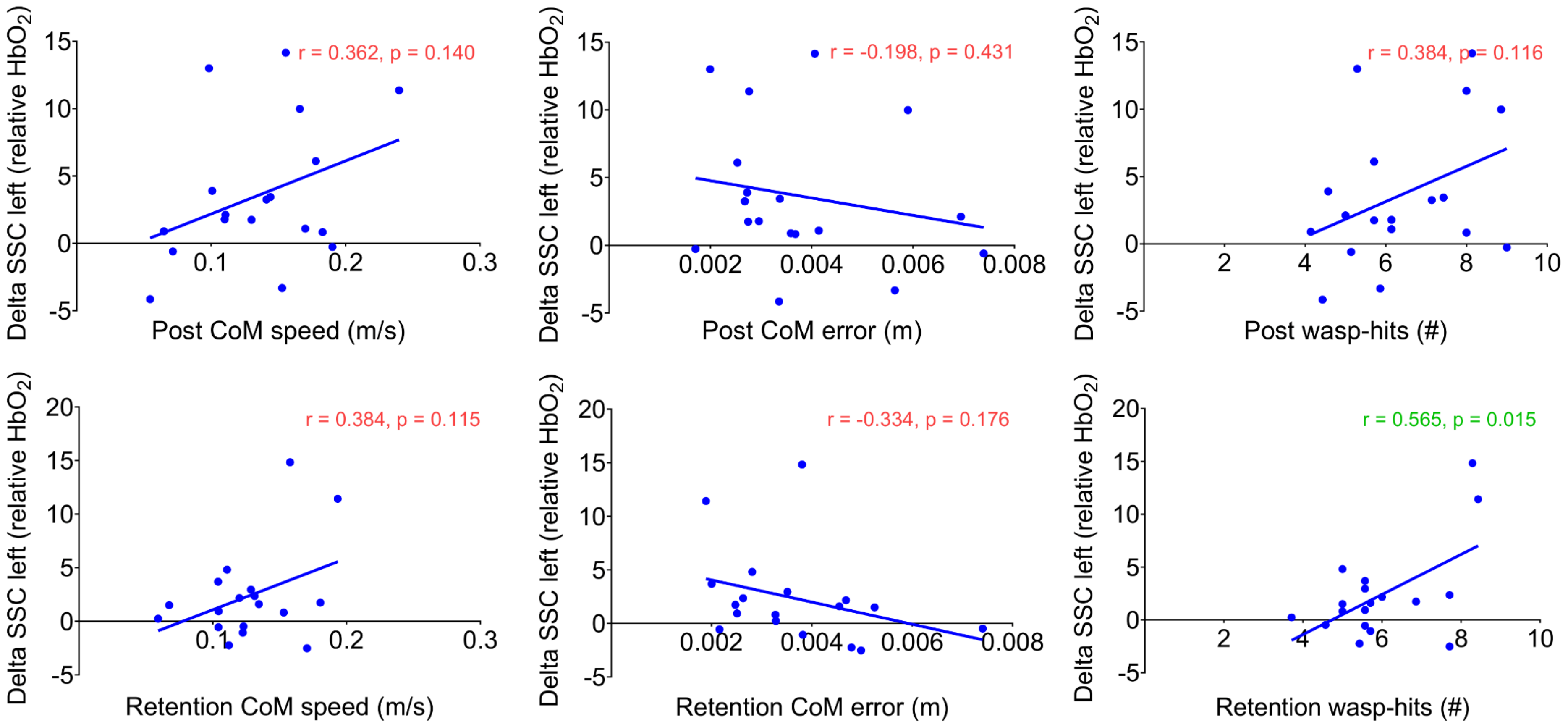


**Supplementary Figure 3.** Correlations between the delta (change) in relative SSC left $HbO_2$ levels from pre- to post-assessment and VR wasp game performance at post-assessment (CoM speed, CoM error, wasp-hits) (top panels), and between the delta (change) in relative SSC left $HbO_2$ levels from pre-assessment to 24h-retention and VR wasp game performance at 24h-retention (CoM speed, CoM error, wasp-hits) (bottom panels). All correlations were performed in the EXP and DT-condition only. CoM = center of mass; SSC = somatosensory cortex; CoM = center of mass; EXP = training group; DT = dual-task.

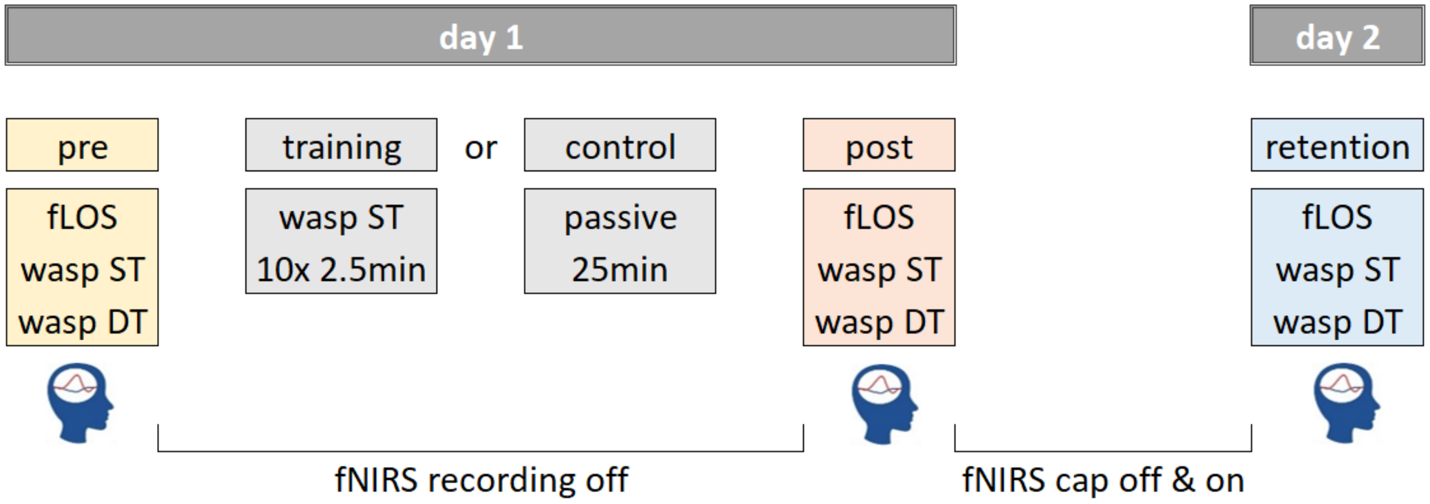


**Supplementary Figure 4.** Procedure of the randomized controlled trial. Training was performed within the virtual reality wasp game. At pre, post and retention, weight-shifting performance was assessed with the functional limits of stability (fLOS) and wasp game in single- (ST) and dual-task (DT) conditions. Cortical hemodynamics were assessed with functional near-infrared spectroscopy (fNIRS).

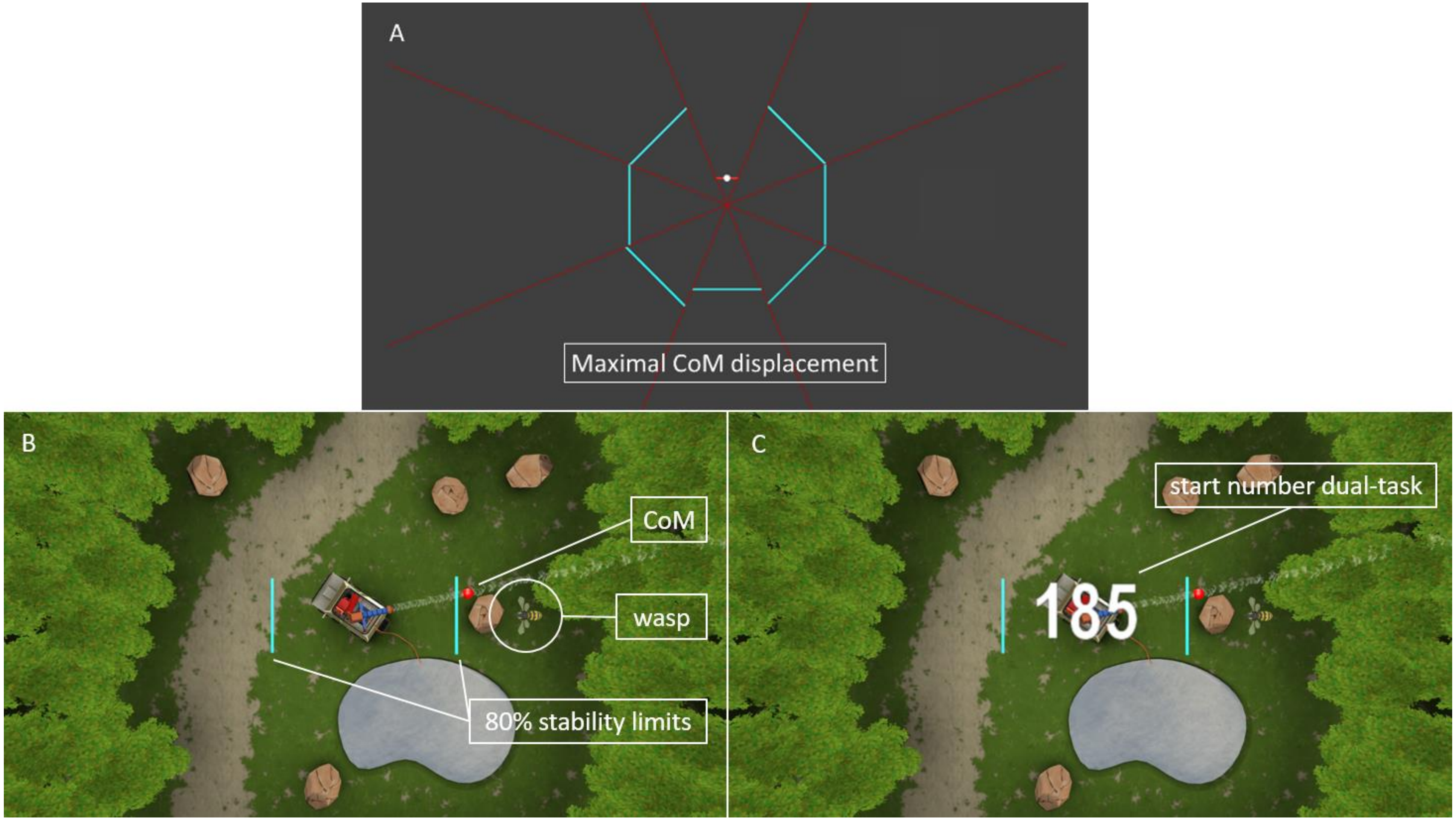


**Supplementary Figure 5.** Visualization of A) the functional limits of stability (fLOS) assessment, B) the virtual reality wasp game in single-task and C) dual-task. The ball could be moved by shifting the center of mass (CoM). Figure modified from de Rond et al. (2021).

de Rond, V., Orcioli-Silva, D., Dijkstra, B. W., Orban de Xivry, J. J., Pantall, A., & Nieuwboer, A. (2021). Compromised Brain Activity With Age During a Game-Like Dynamic Balance Task: Single- vs. Dual-Task Performance. Frontiers in Aging Neuroscience, 13(July), 1–13. https://doi.org/10.3389/fnagi.2021.657308

| | EXP (N=20) | | | CTR (N=20) | | | Group*time |
|---|---|---|---|---|---|---|---|
| | pre | post | retention | pre | post | retention | interaction |
| *ST* | | | | | | | |
| PFC left | 0.34 ± 1.54 | 0.39 ± 1.34 | -0.59 ± 1.96 | 0.64 ± 3.93 | 0.75 ± 4.36 | 1.05 ± 3.60 | 0.234 |
| PFC right | 0.24 ± 1.79 | 0.66 ± 2.66 | 0.27 ± 2.56 | 1.31 ± 3.46 | 0.37 ± 2.24 | 0.72 ± 3.28 | 0.205 |
| FEF left | -0.06 ± 1.60 | 0.56 ± 2.32 | -0.52 ± 2.58 | -0.06 ± 1.48 | 0.22 ± 1.65 | 0.27 ± 2.19 | 0.322 |
| FEF right | 0.25 ± 1.26 | -0.30 ± 2.71 | -0.44 ± 2.23 | 1.20 ± 5.64 | -0.15 ± 2.14 | 0.24 ± 1.91 | 0.529 |
| SMA left | -0.15 ± 2.57 | 0.46 ± 2.59 | -0.26 ± 2.25 | -0.53 ± 4.29 | -0.62 ± 2.69 | -0.12 ± 2.79 | 0.114 |
| SMA right | 0.10 ± 2.13 | 0.24 ± 2.55 | 0.22 ± 2.516 | -0.08 ± 3.65 | -0.86 ± 2.59 | -0.09 ± 2.43 | 0.304 |
| PMC left | -0.65 ± 2.08 | -0.03 ± 2.17 | -0.73 ± 2.45 | 0.14 ± 3.23 | -0.26 ± 3.52 | 0.27 ± 2.63 | 0.096 |
| PMC right | -0.36 ± 1.22 | -0.32 ± 1.81 | -0.20 ± 3.65 | 0.37 ± 1.86 | -0.61 ± 2.18 | 0.01 ± 2.60 | 0.060 |
| SSC left | -0.68 ± 1.09 | -0.63 ± 1.05 | -0.62 ± 1.13 | -0.98 ± 1.66 | -1.22 ± 1.67 | -1.13 ± 1.56 | 0.369 |
| SSC right | -0.58 ± 1.11 | -0.58 ± 1.28 | -1.17 ± 1.28 | -1.01 ± 1.03 | -1.34 ± 1.53 | -1.03 ± 1.18 | 0.188 |
| *DT* | | | | | | | |
| PFC left | -0.02 ± 2.24 | 0.54 ± 1.63 | 0.18 ± 2.05 | 0.16 ± 1.76 | 0.58 ± 3.39 | 0.74 ± 3.01 | 0.765 |
| PFC right | -0.02 ± 1.85 | 1.03 ± 2.51 | 0.84 ± 2.73 | -0.17 ± 2.48 | 0.49 ± 1.99 | 0.70 ± 2.56 | 0.713 |
| FEF left | -0.031 ± 2.04 | 0.05 ± 2.15 | -0.35 ± 1.59 | -0.27 ± 2.45 | 0.65 ± 1.46 | 1.50 ± 3.51 | 0.098 |
| FEF right | 0.17 ± 2.20 | 0.36 ± 2.92 | 0.44 ± 1.99 | 0.49 ± 5.92 | -0.17 ± 2.18 | -0.31 ± 2.45 | 0.508 |
| SMA left | -0.02 ± 2.54 | 0.071 ± 2.45 | -0.16 ± 1.57 | -1.24 ± 4.25 | -0.92 ± 5.04 | 0.72 ± 3.80 | 0.293 |
| SMA right | -0.39 ± 2.09 | 0.28 ± 3.30 | 0.28 ± 1.88 | 0.41 ± 2.42 | 0.02 ± 2.22 | 0.54 ± 2.92 | 0.631 |
| PMC left | -0.83 ± 2.61 | -0.33 ± 2.87 | -0.88 ± 2.10 | 1.21 ± 5.69 | 0.59 ± 6.16 | 0.42 ± 4.00 | 0.193 |
| PMC right | -0.91 ± 1.84 | 0.09 ± 3.05 | 0.63 ± 3.79 | 0.10 ± 2.76 | 0.36 ± 5.04 | 0.06 ± 3.72 | 0.486 |
| SSC left | -0.71 ± 1.09 | -0.66 ± 1.42 | -0.41 ± 1.03 | -0.68 ± 1.16 | -1.12 ± 1.69 | -1.17 ± 1.60 | 0.055 |
| SSC right | -0.812 ± 1.46 | -0.44 ± 1.34 | -1.02 ± 2.34 | -0.87 ± 1.16 | -0.78 ± 1.33 | -0.87 ± 1.80 | 0.688 |

**Supplementary Table 1.** fNIRS HHb levels and interaction effects during wasp game ST and DT.
Values are displayed as mean ± SD (μmol/L). EXP = training group, CTR = control group, ST = single-task. DT = dual-task.
